# Haploid embryos and embryonic stem cells to produce offspring with predetermined parental genomes in cattle

**DOI:** 10.1590/1984-3143-AR2024-0030

**Published:** 2024-08-16

**Authors:** Lawrence Charles Smith, Luis Aguila Paredes, Rafael Vilar Sampaio, Ricardo Perecin Nociti, Jacinthe Therrien, Flavio Vieira Meirelles

**Affiliations:** 1 Centre de Recherche en Reproduction et Fértilité, Faculté de Médecine Vétérinaire, Université de Montréal, Saint-Hyacinthe, Québec, Canada; 2 Laboratório de Morfofisiologia Molecular e Desenvolvimento, Faculdade de Zootecnia e Engenharia de Alimentos, Universidade de São Paulo – USP, Pirassununga, SP, Brasil; 3 Laboratory of Reproduction, Centre of Reproductive Biotechnology – CEBIOR-BIOREN, Faculty of Agriculture and Environmental Sciences, Universidad de la Frontera, Temuco, Chile

**Keywords:** genomics, haploid, embryo, embryonic stem cells, bovine

## Abstract

Selection strategies are performed post-fertilization when the random combination of paternal and maternal genomes has already occurred. It would be greatly advantageous to eliminate meiotic uncertainty by selecting genetically superior gametes before fertilization. To achieve this goal, haploid embryonic cells and embryonic stem cell lineages could be derived, genotyped, and used to substitute gametes. On the paternal side, androgenetic development can be achieved by removing the maternal chromosomes from the oocyte before or after fertilization. We have shown that once developed into an embryo, haploid cells can be removed for genotyping and, if carrying the selected genome, be used to replace sperm at fertilization. A similar strategy can be used on the maternal side by activating the oocyte parthenogenetically and using some embryonic cells for genotyping while the remaining are used to produce diploid embryos by fertilization. Placed together, both androgenetic and parthenogenetic haploid cells that have been genotyped to identify optimal genomes can be used to produce offspring with predetermined genomes. Successes and problems in developing such a breeding platform to achieve this goal are described and discussed below.

## Introduction

Animal selection has traditionally been performed using phenotypic records and pedigrees in which superior animals are chosen as parents according to an estimated breeding value (EBV). Such traditional strategies have been successful for traits with high heritability, e.g. genetic improvement for milk yield, which has increased consistently for many decades. However, progeny testing and pedigree information have proven less effective with traits with low heritability. Moreover, an accurate EBV is costly and time-consuming to obtain due to the recording and analysis of many individuals and the long generation intervals in cattle. Indeed, identifying elite dairy sires by EBV relies on a tedious progeny-testing scheme that takes 6–7 years at substantial costs per bull tested.

Advances in molecular genetics have enabled a novel selection strategy for identifying genetically superior parents by the use of DNA markers associated with quantitative traits (Meuwissen et al., 2001). A key breakthrough in marker-assisted selection came with the sequencing of the whole bovine genome (Gibbs et al., 2009), which has led to the discovery of many thousands of DNA markers in the form of single nucleotide polymorphisms (SNP) associated with production traits. These novel molecular tools have dramatically reduced the cost of genotyping. A second breakthrough came with the demonstration that it is possible to make accurate selection decisions when breeding values are predicted from DNA markers alone by calculating genomic breeding values (GEBV). Moreover, the implications of achieving accurate GEBV for animals at birth are profound. Potentially, genomic selection can lead to a doubling of the rate of genetic gain through selection and breeding from bulls at 2 years of age rather than 5 years of age or later (Schaeffer, 2006). Although more genotyping is needed to increase selection intensity and thereby increase the rates of genetic gain, it is expected that cattle breeding companies can save a large majority of their costs using GEBV instead of the traditional EBV (Hayes et al., 2009). Genetic gain can also be improved by employing genomic selection strategies in combination with advanced reproductive technologies and the largest increase in genetic gain can be achieved by shortening the generation interval and it is now possible to evaluate the genetic merit of a newborn calf or even a pre-implantation stage embryo (provided that a reference population is available) with comparable accuracy at a much-reduced cost (Georges, 2014).

## Meiotic segregation is an uncontrolled source of variability

Due to the random nature of meiosis, one can never accurately predict which set of parental genes will be transmitted by each gamete to the offspring. Although cattle generation intervals can be dramatically decreased and selection accuracy can be greatly improved by using the genomic approach and selecting genetically superior offspring very early post-fertilization, selection programs are consistently limited by independent assortment and crossing over of parental chromosomes during meiosis, causing uncontrollable genomic variability before fertilization. Genetic diversity is ensured during two meiotic events, i.e. crossing over and independent assortment of chromosomes. Crossing over occurs during prophase I of meiosis and enables homologous pairs of chromosomes to recombine and often exchange chromosome segments. This allows genes from each parent to intermix and create chromosomes with a different genomic complement. Independent chromosome assortment occurs during meiosis II when sister chromatids separate and are randomly distributed to the daughter cells, i.e. gametes. In cattle, independent assortment can yield 2^30^, or 1,073,741,824, unique ways to arrange 30 pairs of chromosomes.

**Figure 1:**
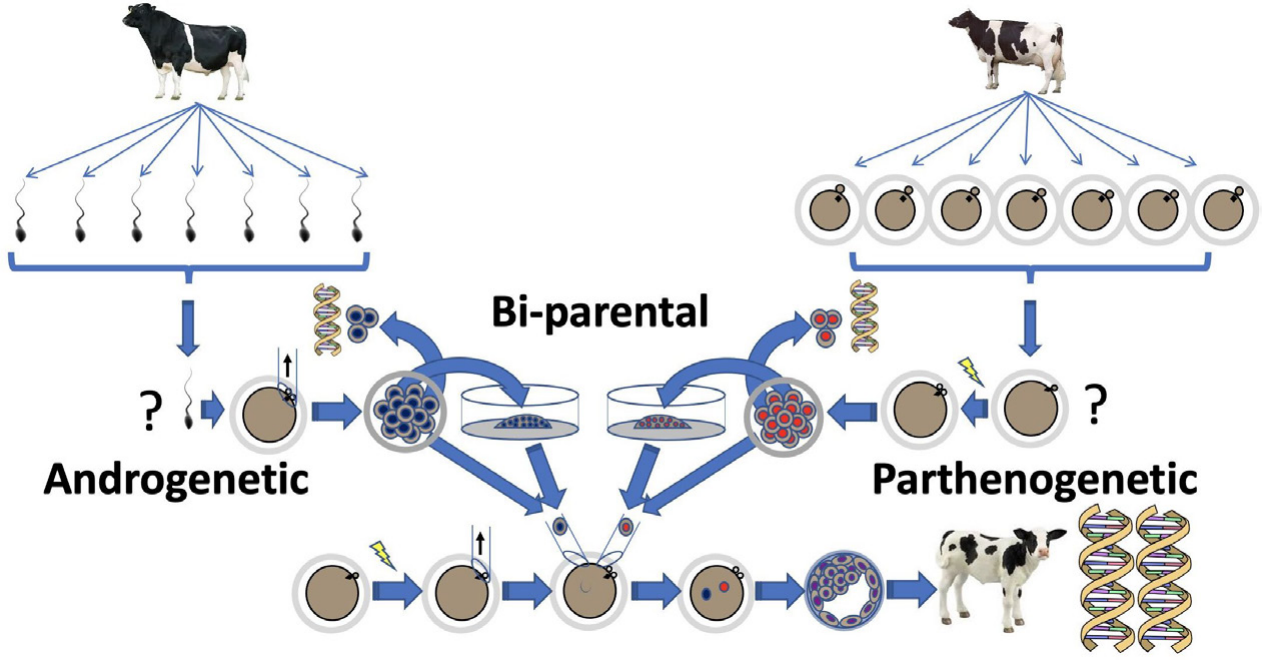
Strategy to produce cattle with predetermined genomes by reconstructing zygotes with genotyped haploid embryonic cells of androgenetic, parthenogenetic, or bi-parental origin.

To date, selection strategies are performed post-fertilization when the random combination of paternal and maternal genomes has already occurred. It would be greatly advantageous to eliminate meiotic uncertainty by selecting genetically superior gametes before fertilization. Therefore, we believe that haploid cells derived from sperm (paternal) and oocytes (maternal) can be obtained by androgenesis and parthenogenesis, respectively, and then genotyped to select those carrying superior genomic markers “beforehand” so that only the most promising haploid cells be used to ‘reconstruct‘, i.e. fertilize, zygotes, embryos and offspring with predetermined optimal genomes (Figure 1).

## Derivation and developmental outcome of bovine haploid embryos

Diploid genomes (two sets of chromosomes, one maternal and one paternal) are typical among most living animals, and haploidy (a single set of chromosomes) is generally limited to gametes. Although a diploid genome is thought to increase fitness by masking mutations, it also leads to the accumulation of mutations with time. To counteract long-term genome degradation, mammals created adaptations that include genomic imprinting, random monoallelic expression, and X chromosome inactivation (Wutz, 2014). Haploidy is normally restricted to the post-meiotic stages of germ cells and represents the end point of cell proliferation, which means that physiological haploidy is incompatible with self-renewal.

To date, few studies have been performed to study androgenetic development in cattle. The first report came from Galli’s group which produced haploid and diploid androgenotes by in vitro fertilization (IVF) and intracytoplasmic sperm injection (ICSI) of enucleated metaphase-arrested (MII) oocytes. Although cleavage rates were similar to controls, haploid embryos developed poorly to the morula stage and most arrested before reaching the blastocyst stage (Lagutina et al., 2004). These results were confirmed later in androgenotes produced before and after oocyte showing a small advantage of the former group (Vichera et al., 2011). Our experiments showed that when using IVF, regardless of whether enucleation was performed before or after fertilization, the levels of polyspermic fertilization occur quite often as indicated by the identification of 2 or more pronuclei after fixations and DNA staining at 20 h after fertilization even after shortening the exposure to spermatozoa to 6 h during IVF. In contrast, when using ICSI followed by removal of the oocyte’s spindle during telophase, polyspermy was no longer an issue since zygotes with a second polar body contained only a single pronucleus (Aguila et al., 2021).

**Figure 2:**
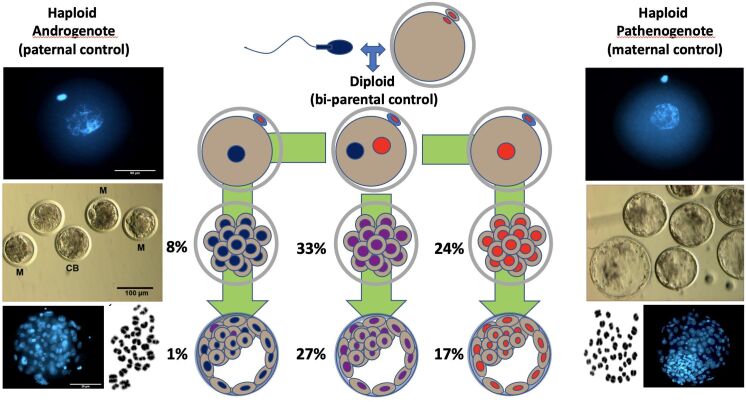
Schematic outline of diploid (ICSI-derived) androgenetic and parthenogenetic haploid embryos and their developmental outcomes (in percent) to the morula stage (day 6; middle row) and blastocyst stage (day 7; bottom row). Representative images of androgenetic (left) and parthenogenetic (right) zygotes (pronuclei), embryos (light microscopy), nuclear staining, and karyotype at day 7.

Once the reliability of the ICSI approach for deriving hAE was verified, we compared early developmental rates of haploid and diploid control groups at different times of in vitro culture (Aguila et al., 2021). As reported in mice (Latham et al., 2000, 2002), our studies in cattle showed that androgenetic embryos produced using Y-chromosome sperm rarely support development beyond the 8-cell stage whereas when using X-chromosome sperm 9% develop to the morula and 3% to the blastocyst stage, respectively. However, compared to ICSI and haploid parthenotes, haploid androgenotes produced using X-bearing sperm show significantly lower development to the morula and blastocyst stage as measured on days 6 and 7 of in vitro culture, indicating that the paternal genome fails to provide appropriate conditions for normal development and differentiation beyond embryonic genome activation (EGA). In contrast, haploid parthenotes produced by the activation of metaphase-arrested oocytes that extrude a second polar body after ionomycin (5 min) fand cycloheximide (4 h) exposure develop well to the morula stage (24% vs. 31%) and, although cell numbers are similar, slightly less well to the blastocyst stage (16% vs. 26%) when compared to diploid ICSI embryos. Together, these results suggest that the maternal genome is more effective than the paternal in supporting development beyond EGA when in a haploid condition (Figure 2). However, compared to the androgenotes that showed consistent haploidy (30 chromosomes) at the morula stage, parthenotes had a much variable chromosomal number, with only a third showing haploidy, while the remaining contained either 60 chromosomes (diploid) or were aneuploid with intermediate chromosome counts, indicating complete and/or partial diploidizations. Aneuploidies and mixploidies have been previously reported in porcine and bovine parthenotes (Cheng et al., 2007; Winger et al., 1997). Although the causes of aneuploidy in haploid parthenotes remains unknown, one possibility is that haploid blastomeres fail to undergo cytokinesis or that they fuse after cleavage. Another possibility is that paternal centrioles may be essential to obtain proper chromosomal segregation during the initial mitotic divisions. Humans, pigs, and cows are among the numerous mammalian species that lack maternal centrioles in mature oocytes centrioles (Navara et al., 1994; Sathananthan et al., 2006; Schatten et al., 1991), which may explain why parthenotes develop aneuploidies. In contrast, bovine androgenotes maintain stable chromosomal haploidy throughout early development, which may be due to the presence of the paternally inherited centrosome carried into the oocyte at fertilization.

## Transcriptional patterns of bovine haploid embryos

**Figure 3:**
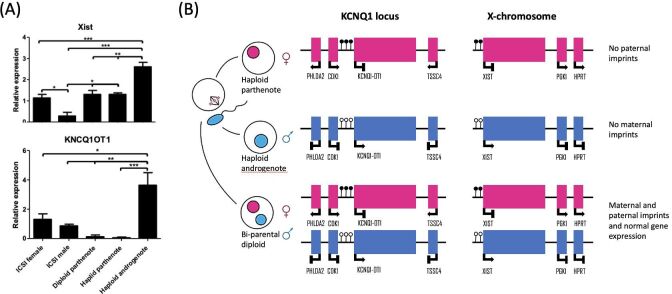
Imprinting and X-chromosomal expression in parental-specific fashion. (A) Non-coding transcripts responsible for the inactivation of X-chromosome (XIST) and the imprinted gene loci KCNQ1 (KCNQ!OT1) are significantly up-regulated in androgenetic embryos compared to parthenogenetic and biparental (ICSI) morula-stage embryos; (B) Schematic of the mechanisms involved in regulating the KCNQ1 locus and of the X-chromosome according to parental origin.

To further our understanding of the causes for the poor development to blastocysts of haploid X chromosome-bearing androgenetic (3%) and parthenogenetic (16%) embryos when compared to diploid female ICSI (26%) embryos, transcriptional characterization was first aimed at identifying differences in parental-specific transcripts of imprinted genes and the X-chromosome (Figure 3). Indeed, after analyzing the transcripts of genes from the X-chromosome, we found an overexpression of XIST (for X-inactive specific transcript), the non-coding RNA transcribed from the X-chromosome in mammals that acts as a major effector of the X-inactivation process. These results indicate that the XIST gene on the paternal X-chromosome is preferentially activated at the morula stage and could be responsible for downregulating the expression of X-chromosome genes responsible for early embryonic differentiation leading to the poor development beyond the EGA in haploid X chromosome-bearing androgenotes. Moreover, in contrast to the maternally imprinted genes IGF2R and GNAS, androgenetic embryos showed overexpression of KCNQ1OT1, the long non-coding RNA (lncRNA) that controls the imprinted KCNQ1 domain localized on the bovine chromosome 29. Interestingly, methylation patterns of the differentially methylated regions regulating XIST and KCNQ1OT1 expression were consistent with the parental origin of the allele, suggesting methylation of such DMRs cannot explain the overexpression of these lncRNA in the haploid androgenotes (Aguila et al., 2021).

**Figure 4:**
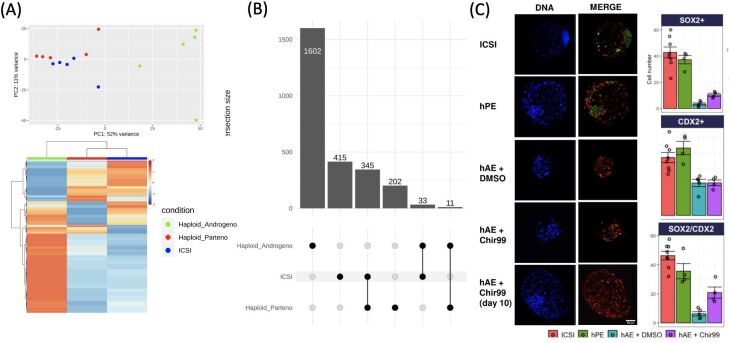
Transcriptomic patterns and pluripotency markers in haploid androgenetic, parthenogenetic, and diploid ICSI embryos. (A) Principal component analysis (PCA) plot and heatmap of differentially expressed genes (DEG); (B) UpSet plot illustrating each group’s DEG number and intersections between transcriptomes; (C) Representative immunofluorescent images (red=CDX2; green=SOX2) of blastocysts and cell numbers for each category, including an androgenetic group exposed or not (DMSO vehicle) to CHIR99021, a GSK3ß inhibitor.

Having analyzed the transcriptional patterns of imprinted domains and X-chromosome activity in haploid embryos, we next focused our attention on performing a global assessment of the transcriptome of haploid embryos using RNAseq (Aguila et al., 2023). As observed by principal component analysis (PCA) and by the heatmap of the differentially expressed genes (DEG), we observed an isolated clusterization and diverse pattern of the haploid androgenotes in comparison to the diploid (ICSI) and parthenotes. In contrast, diploid and parthenotes clustered together and showed similar heatmap patterns (Figure 4). Numerically, androgenetic genes contained four times more and eight times more DEG than ICSI and parthenotes, respectively. Immunofluorescent assessment of ICM (SOX2) and TE (CDX2) markers indicated that the androgenotes had fewer total cells and that the difference was exacerbated by fewer ICM cells. However, exposure to the CHIR99021, a specific inhibitor of GSK3ß, led to a significant increase in the SOX2/CDX2 ratio, suggesting that the WNT signaling pathway is involved in the impaired cell fate of androgenotes (Figure 4).

## Use of haploid embryonic cells for parental replacement

**Figure 5:**
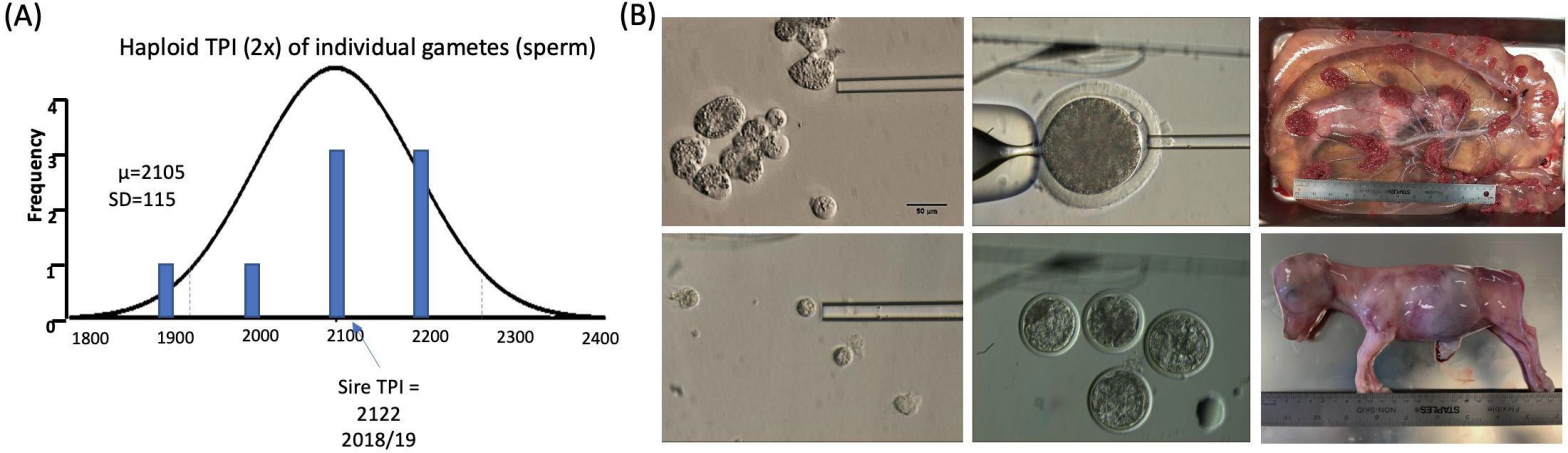
Reconstruction of diploid zygotes, embryos, and fetuses using androgenetic embryonic cells to replace spermatozoa. (A) Haploid total performance index (TPI) of genomes from individual sperm as compared with the TPI of the sire; (B) Images of individualized haploid donor cells obtained from morula and embryonic outgrowths, reconstruction of a diploid zygote, and representative examples of blastocysts, day-100 conceptus, and fetus derived from diploidized zygotes.

Further research is required to improve the development of androgenetic and parthenogenetic embryos to enable the multiplication of haploid cells of characterized genomes to enable storage and continuous usage to obtain offspring with predetermined genomes of paternal, maternal, or biparental origin. Nonetheless, having shown that haploid cells of androgenetic origin could be obtained reliably from morula stage embryos, we undertook experiments to reconstruct diploid embryos by injection of haploid blastomere nuclei into parthenogenetically activated oocytes (Figure 5). Haploid androgenetic blastomeres were isolated and genotyped to determine their haploid genomic value, which enabled the identification of haploid embryos with genomes ranked above the average genomic value of the sire. As a proof of concept, these results indicate that haploid embryonic cells can be genotyped using appropriate genomic evaluation pipelines.

Although cleavage rates were not affected compared to ICSI controls, significantly fewer cleaved embryos reached the 4- to 8-cell stage by 48h, indicating a delayed mitotic activity during early development. Moreover, fewer reconstructed embryos developed to the morula stage at day six and to the blastocyst stage at day seven of in vitro culture. Together, these results show a retarded development of zygotes reconstructed using haploid androgenetic nuclei, indicating that the developmental abnormalities described in the haploid embryos may interfere with the early development of the diploid reconstructed zygotes. Nonetheless, although the ratio of inner-cell-mass/trophectoderm cells was lower in reconstructed blastocysts after freezing and thawing, the total number of nuclei was not different from ICSI controls. Twelve reconstructed blastocyst stage embryos were transferred to synchronized recipients resulting in 4 gestations at 60 days. Although fewer recipients transferred with reconstructed embryos remained pregnant at Day 60, crown-rump length ultrasound measurements at Day 30 of pregnancy did not differ with control gestations, indicating that post-implantation development at early stages of gestation did not differ from controls. To further evaluate *in vivo* development at later stages post-transfer, reconstructed conceptuses were collected at slaughter between 92 and 106 days after transfer. Morphological measurements of conceptuses from control and treatment groups showed similarities in fetal size, body weight, and organ weight, indicating that reconstructed embryos show characteristics of viable gestations.

## Development of haploid embryonic stem cells in mammals

The advent of haploid mouse embryonic stem cell (ESC) technologies opens opportunities for numerous fields (Kokubu and Takeda, 2014). Recently, several studies have derived ESCs from mammalian parthenogenetic and androgenetic haploid embryos. Initial studies were focused on mice (Elling et al., 2011; Leeb and Wutz, 2011; Li et al., 2012; Yang et al., 2012); however, similar techniques have also been applied to monkeys (Wang et al., 2018; Yang et al., 2013) and rat haploid ESC derivation (Li et al., 2014). The original versions of haploid ESC lines (Elling et al., 2011; Leeb et al., 2014) were generated by parthenogenetic activation of unfertilized mouse oocytes with chemicals such as strontium salt or ethanol. These haploid mouse ESCs contain only the maternal set of chromosomes, show pluripotency and self-renewal capabilities. Androgenetic haploid mouse ESC lines containing only the paternal chromosomes have also been generated by removing the maternal pronucleus from zygotes and introducing sperm into enucleated oocytes (Li et al., 2012; Yang et al., 2012). Thus, pluripotency, self-renewal, and haploidy can be incorporated together in a single cell line. Future studies aimed at and succeeded in deriving parthenogenetic and androgenetic haploid embryonic stem cells in humans (Sagi et al., 2016, 2019; Zhang et al., 2020; Zhong et al., 2016).

Haploid ESC lines have been shown to function as gametes and support further embryonic development (Li et al., 2012; Shuai and Zhou, 2014; Wan et al., 2013; Yang et al., 2012). Metaphase oocytes were ‘fertilized’ with haploid ESC by intracytoplasmic cell injection resulting in producing fertile pups. Although most of the pups developed to adulthood and gave birth to the next generation, some newborn pups died shortly after birth due to developmental retardation, suggesting an abnormal imprinting state of donor haploid ESCs (Li et al., 2012; Yang et al., 2012). In another experiment sperm were injected into an enucleated oocyte, followed by the activation of the reconstructed embryos by chemical stimulus. Pups were generated, albeit at low efficiencies, suggesting either loss of imprinting of haploid ESCs or effects of enucleation during zygote reconstruction (Wan et al., 2013). Therefore, maintenance of maternal and paternal imprints is key in enabling the normal development of androgenetic and parthenogenetic haploid ESC lines (Hu et al., 2015; Li et al., 2014, 2016; Yang et al., 2012), highlighting the importance of verifying the imprinting status of haploid ESC lines when developing strategies to generate viable offspring. The occurrence of spontaneous diploidization has been a major hurdle in the application of haploid embryonic cells. Although the mechanisms by which haploid cells undergo diploidization remain uncertain, it has been proposed that it may result from either nuclear re-replication or cell fusion (Kokubu and Takeda, 2014) and the use of cell sorting together with the exposure to molecules that control the cell cycle has become an important strategy to overcome the gradual propensity for diploid cells to overtake the haploid cell population (He et al., 2017; Li et al., 2017; Takahashi et al., 2014).

## Derivation of bovine haploid embryonic cells and their potential use for parental replacement experiments

**Figure 6:**
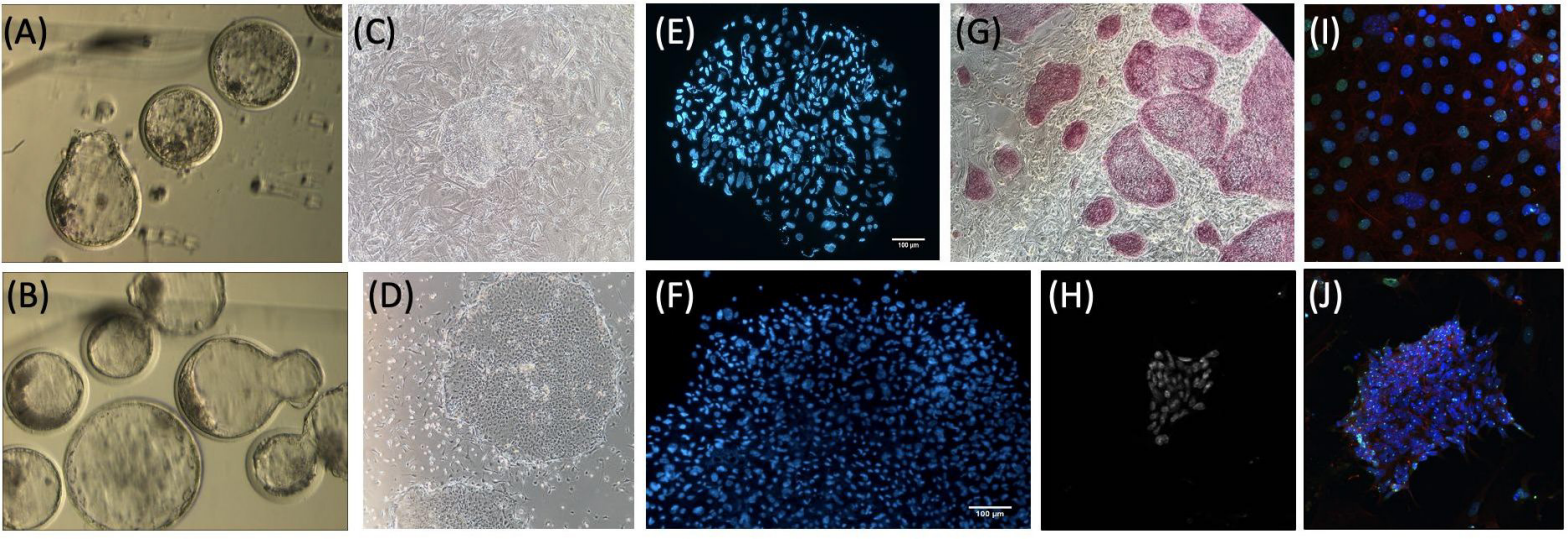
Establishment of haploid embryonic stem (ES) cell-like lineages from haploid (A) androgenetic and (B) parthenogenetic blastocysts. Haploid parthenogenetic colony outgrowth at (C) Day-5 with (E) around 300 nuclei and at (D) Day-7 with approximately 4000 nuclei. Positive staining for (G) alkaline phosphatase and (H) OCT4. Immunostaining for H3K27me3, indicative of the presence of Barr body, was (I) absent at P0 and (J) positive (arrows) at P5 in established haploid parthenogenetic ES lines.

Several groups have reported the derivation of diploid embryonic stem cell-like cells (Cao et al., 2009; Cibelli et al., 2002; Pashaiasl et al., 2010; Singh et al., 2012). However, to our knowledge, no previous report has focused on the derivation of haploid ES-like lines in cattle. Our unpublished results have focused on using haploid blastocysts derived by androgenesis and parthenogenesis. Parthenotes were produced using a protocol involving 5 min exposure of secondary oocytes to ionomycin followed by a 3-hour exposure to either cycloheximide (CHX) or anisomycin (ANI), protocols which produces between 10 to 15% blastocysts. As a control, we utilized diploid parthenotes derived by adding cytochalasin B to block second polar body extrusion during activation producing between 30% and 40% blastocysts. Approximately 95% of the haploid blastocysts attach within one day of plating and 30% establish an outgrowth with approximately 300 cells on Day 5 and 4000 cells on Day-7 of culture (Figure 6). Seventy percent of the outgrowth colonies (P0) are viable and can be passaged (P1) a week after plating. Moreover, most lines that can be maintained to passage five or beyond are positive to alkaline phosphatase and OCT4 immunostaining, indicating pluripotency. However, analysis of the established parthenogenetic lines derived from haploid parthenotes showed the presence of a Barr body, as indicated by immunostaining to H3K27me3 in the nucleus of most cells. These results indicate the presence of an inactive X-chromosome and possibly the duplication of the single X-chromosome present in haploid cells. Since previous karyotypic analysis of parthenotes had already shown a high percentage of aneuploid cells in haploid parthenogenetic embryos (Aguila et al., 2021). Therefore, we performed FACS and karyotype analysis in haploid parthenogenetic lines at different passages and showed a large percentage of aneuploid and diploid cells, suggesting a progressive diploidization of haploid parthenogenetic lineages during in vitro culture and passaging. Experiments are currently being performed to develop strategies that revert and/or avoid such diploidizations.

## Conclusions

Although the derivation of haploid embryonic stem cell lines remains challenging in many species, major hurdles remain for producing stable lineages of both androgenetic and parthenogenetic in cattle. On the androgenetic side, success in obtaining viable conceptuses from haploid androgenetic morula-stage shows that it is possible to multiply the male gamete, i.e. sperm, to a level where genomic evaluations can be performed to decide on whether it is desirable as a paternal genome for the forthcoming offspring. Although initial attempts to produce embryos that developed to the blastocyst stage, current protocols have improved blastocyst outcomes to a level in which embryonic outgrowths and early passage ES-like cells can be consistently obtained. Moreover, although haploid male lines remain challenging due to the lack of the X-chromosome, attempts to produce Y-bearing early haploid embryos have shown that the development to the early morula stage can be achieved, which enables genomic characterization and diploidizations to produce a limited number of male offspring with predetermined paternal genomes. Although development to blastocyst and ES-like lines can be readily achieved, diploidizations and aneuploidies remain a significant challenge in obtaining haploid cells for deriving viable offspring. As shown in the derivation of haploid ES lines in mice and humans, FACS sorting of haploid cells may be the best option together or not with the use of culture conditions that are more permissive to maintain haploidy.
